# Neutrophil Gelatinase‐Associated Lipocalin and Neutrophil‐to‐Lymphocyte Ratio as Indicators of Nephropathy in Type 2 Diabetic Mellitus Patients in Ghana: A Case‐Control Study

**DOI:** 10.1002/hsr2.71939

**Published:** 2026-03-01

**Authors:** Allwell Adofo Ayirebi, Wina Ivy Ofori Boadu, Stephen Twumasi, Samuel Kwarteng, Benedict Sackey, Lilian Antwi Boateng, Joseph Boachie, Samuel Essien‐Baidoo, Daniel Nii Martey Antonio, John Agyemang Sah, Afua Marfowaa, Mayfair Adwapa Mpiani, Joachim Baba Domosie, Abiba Khalifah, Emmanuel Ekow Korsah, Ebenezer Senu, Eugene Arele Ansah, Joseph Yorke, Enoch Odame Anto

**Affiliations:** ^1^ Department of Medical Diagnostics, Faculty of Allied Health Sciences, College of Health Sciences Kwame Nkrumah University of Science and Technology Kumasi Ghana; ^2^ Department of Molecular Medicine, School of Medicine and Dentistry Kwame Nkrumah University of Science and Technology Kumasi Ghana; ^3^ Department of Medical Laboratory Science University of Cape Coast Cape Coast Ghana; ^4^ University of Health and Allied Sciences Ho Ghana; ^5^ Department of Surgery, School of Medicine and Dentistry Kwame Nkrumah University of Science and Technology Kumasi Ghana; ^6^ School of Medical and Health Sciences Edith Cowan University Perth Western Australia Australia; ^7^ Centre for Precision Health, ECU Strategic Research Centre Edith Cowan University Perth Western Australia Australia

**Keywords:** diabetic nephropathy, microalbuminuria, neutrophil‐to‐lymphocyte ratio, serum neutrophil gelatinase‐associated lipocalin, type 2 diabetes mellitus

## Abstract

**Introduction:**

Serum neutrophil gelatinase‐associated lipocalin (sNGAL), a renal tubular marker, and neutrophil‐to‐lymphocyte ratio (NLR), a hematological inflammatory marker are two biomarkers that have recently received attention, because of their association with kidney disease. This study examined the diagnostic value sNGAL and NLR in type 2 diabetes mellitus (T2DM) patients with nephropathy.

**Materials and Methods:**

In this hospital‐based case‐control research, 97 T2DM participants and 70 healthy subjects were included. Participants' information was documented using a structured questionnaire and patient case records. Venous blood was drawn from each participant and early morning midstream urine samples were collected for blood and urine measurements respectively.

**Results:**

The prevalence of nephropathy among type 2 diabetics was 20.6%. sNGAL had a good performance (AUC = 0.793, *p* < 0.001) and NLR had a poor performance (AUC = 0.632, *p* = 0.082) for predicting diabetic nephropathy. An sNGAL cut‐off of 8.87 µg/L had 80.0% sensitivity and NLR threshold of 2.34 had 60.0% sensitivity and 72.8% specificity. In the multivariate binary logistic model, sNGAL showed a modest independent association, (aOR 1.25, 95% CI: 0.71–2.21, *p* = 0.014). In contrast, NLR was not significantly associated with nephropathy in the adjusted model (NLR: aOR 1.16, 95% CI: 0.60–2.24, *p* = 0.663).

**Conclusions:**

Whiles sNGAL showed superiority and is recommended, NLR showed no significant group difference and poor, non‐significant discrimination, indicating it is not a reliable standalone marker of diabetic nephropathy in this study.

## 1 Introduction

Type 2 diabetes mellitus (T2DM) is a metabolic illness that affects most people worldwide. Globally, the prevalence of diabetic mellitus (DM) is believed to be at 8.5%, and it is predicted that this number could double in the next 10 years [1]. Numerous research has indicated that the prevalence of diabetes mellitus in Ghana varies from 6.4% to 13.9% [2, 3].

Diabetic nephropathy (DN), as a major vascular adverse outcome of DM, arises due to uncontrolled hyperglycemia resulting in glomerular and tubular destruction and eventual permanent kidney damage [4]. A lower estimated glomerular filtration rate (GFR) of less than 60 mL/min/1.73 m^2^ and albuminuria for a minimum of 3 months are indicative of chronic kidney disease [5]. The prevalence of diabetic nephropathy in Ghana ranges from 14% to 19% [6]. A 13‐year retrospective study found diabetic nephropathy to be the third leading cause of chronic kidney disease, and the second leading cause of end‐stage kidney disease in Ghanaian adults [7].

In diabetic adults, microalbuminuria—generally characterized as a urine albumin excretion rate of 30–300 mg per 24 h—is recognized as an independent indicator of an elevated risk for renal and cardiovascular disease [8]. Progressive albuminuria is a well‐accepted criterion for assessing the severity of diabetic nephropathy, while microalbuminuria is a sign of the early stages of diabetic nephropathy in diabetes mellitus [9, 10]. The conventional estimated Glomerular Filtration Rate (eGFR) and its albuminuria correlation (UACR) are regarded as the primary laboratory markers for indicating chronic kidney disease (CKD) in Ghana. However, creatinine is a marker of glomerular filtration and is unable to appropriately assess renal tissue damage. Also, serum creatinine rises after about 50% of kidney function is lost, hence not sensitive enough to pick up on subtle variations in renal function that might occur before albuminuria manifests [11, 12]. It has been widely proposed that other biomarkers are needed for early detection of renal dysfunction. There is also the need for cost‐effective and accessible biomarkers that can effectively detect DN, as current markers are expensive and financially burdensome for both the government and individuals [13].

A tubulointerstitial injury marker, neutrophil gelatinase‐associated lipocalin (NGAL), is a 25 kDa protein that stems from the lipocalin superfamily has been suggested as a potential marker of CKD [14]. It is mainly used to transport tiny hydrophobic molecules and control innate immune responses. It is secreted by renal tubular epithelial cells and released by active neutrophils [15]. The hematological inflammatory neutrophil‐to‐lymphocyte ratio (NLR) measures the interaction between the innate and adaptive cellular immune responses. NLR is an affordable and easily measurable marker of inflammation, indicating inflammatory burden in many chronic conditions. It has been shown that NLR can serve as an early marker for DN, evidenced by a lower predicted risk of hospitalizations in diabetic patients undergoing hemodialysis [16]. The efficacy of these two markers in detecting DN has not been evaluated in the context of Ghana. As a result, the study assessed the performance of sNGAL and NLR in predicting nephropathy among type 2 diabetics. Moreover, given the major role uncontrolled hyperglycemia plays in diabetic nephropathy incidence, this study also assessed these markers in DN with good and poor glycaemic management.

## 2 Materials and Methods

### 2.1 Study Design, Duration, and Study Setting

This was a prospective case‐control study and conducted at the Ejisu Government Hospital from December 15, 2022 to February 25, 2023. The Ejisu Government Hospital, located in the Ejisu Juaben Municipality of the Ashanti region of Ghana, served as the site of this hospital‐based case‐control study. The hospital is the biggest healthcare facility in the municipality, providing an extensive array of services including emergency medical treatment, obstetrics and gynecology, laboratory testing, and medical imaging. Participants were chosen for the study from the facility's diabetic clinic.

### 2.2 Ethical Clearance and Informed Consent

The Committee for Human Research Publication and Ethics of Kwame Nkrumah University of Science and Technology, School of Medical Sciences, evaluated and approved the study protocol, permission forms, and participant information material (CHRPE/AP/742/22). The Ejisu Government Hospital ethical committee also approved the study. Once the study participants had received a thorough explanation of the study's objectives, advantages, risks, and right to withdraw at any time in both English and the local dialect (primarily Twi), their written consent was requested.

### 2.3 Study Participants

A total of 167 participants were recruited for this study. Cases consisted of 97 type 2 diabetics (21 males and 76 females) under management at the clinic, 6 months or more prior to recruitment of study, and 70 participants without diabetes (28 men and 42 females) used as healthy controls. Physicians in the hospital assessed the diabetic patients and declared them fit for the study before they were recruited. Patients with cancer, microbial infections, pregnant women, cardiovascular conditions, and advanced kidney impairment (eGFR < 30 mL/min) were excluded. Additionally, we excluded diabetics with iron deficiency anemia, HIV infection, hepatitis C, and inherited hemolytic disorders. Moreover, participants with acute alterations in white blood cell count—defined as values outside the reference range (4.5–11.0 × 10⁹/L) [17] in conjunction with clinical evidence of acute infection or inflammation were excluded. Patients with stable but chronically elevated counts, such as those related to chronic disease, were included.

### 2.4 Data Collection

Demographic and clinical history of the participants were recorded using well‐structured questionnaires and patient case records. Dietary habits were categorized qualitatively as occurring rarely (one per month), irregularly (seven times per month), and regularly (twice per month).

Estimates were made for height to the nearest centimeter without shoes and weight to the nearest 0.1 kg when wearing light clothes. A wall‐mounted ruler was used to measure height. A Zhongshan Camry Electronic Co. Ltd. bathroom scale (Guangdong, China) was used to assess weight.

### 2.5 Blood and Urine Sampling

After an 8–12 h overnight fast, venous blood sample was drawn from each participant into fluoride/oxalate, serum separator tube (SST) and ethylenediamine tetraacetate (EDTA) tubes. After clotting, the SST was centrifuged and kept at −20°C until analysis. Early morning midstream urine samples were used for urine measurements.

### 2.6 Laboratory Assays

#### 2.6.1 Blood Measurements

Hemogram analysis of the EDTA samples were determined within 1 h after sample using an automated hematology analyzer (XN‐550; Sysmex Corporation, Kobe Japan). NLR levels were determined from hemogram test by calculating the ratio of absolute neutrophil count and absolute lymphocyte count. Glycosylated hemoglobin test was run on a Getein Biotech Immunofluorescence Quantitative Analyzer (Getein 1100, China). Well‐controlled diabetes and poorly controlled diabetes was defined as HbA1c ≤ 7% and > 7% respectively. The Mindray BS‐240 fully automated chemistry analyzer and an assay kit (Shenzhen Mindray Bio‐Medical Electronics Co., China) were used to estimate the plasma concentration and level of creatinine in serum samples. The estimated Glomerular Filtration Rate (eGFR) was calculated using the CKD‐EPI equation from [18].

A commercially available ELISA kit (Melson Shanghai Chemical Ltd., China) was used for the sNGAL measurement. The reagent was used in accordance with the manufacturer's instructions to measure samples from the controls and the participants using the solid phase ELISA technique.

#### 2.6.2 Urine Culture

Urine cultures were used as a screening method to weed out people who had urinary tract infections (UTI). A 10 µL aliquot of each participant's urine was inoculated onto cystine‐lactose‐electrolyte deficient (CLED) agar using a sterile 10 µL inoculation loop, and was then incubated for 24–48 h in an aerobic environment [19]. Participants having a colony count more than 1000 colony‐forming units per milliliter (CFU/mL), which indicates a significant urinary tract infection, were eliminated from the study after the resulting cultures were assessed.

#### 2.6.3 Microalbuminuria Measurement and Definition of Diabetic Nephropathy

The urine albumin‐to‐creatinine ratio was analyzed on a Vitros 5600 integrated system following standard protocols [20, 21]. In accordance with the 2024 KDIGO classification [22] for establishing chronic kidney disease, the UACR was measured on three separate occasions within 6 months for both diabetic and non‐diabetic participants. Participants were diagnosed with microalbuminuria if their UACRs measured three times were greater than 30 mg/g [23]. Individuals with only one or only two UACR measurements greater than 30 mg/g were considered to have probable microalbuminuria and excluded. Only individuals with three consecutive UACR measurements greater than 30 mg/g were classified as having microalbuminuria, and those with three consecutive UACR measurements less than 30 mg/g were classified as having non‐microalbuminuria. The average of the three UACRs measured was used for analysis. Also, diabetic nephropathy was defined as individuals with microalbuminuria (UACR > 30 mg/g measured on three occasions). UACR was used as a gold standard of which other markers were compared to.

### 2.7 Statistical Analysis

The data obtained from the study was entered into Microsoft Excel software (2016) and analysed using R programming version 4.2.3, XLSTAT (v2019.2.2) and IBM Statistical Package for Social Sciences (version 26.0). In contrast to categorical variables, which were expressed as proportions and compared using the *χ* ^2^ test, parametric continuous variables were expressed as mean standard deviation. Also, non‐parametric variables were expressed as median and interquartile range. The *χ* ^2^ test was used to examine the relationship between categorical research variables and diabetics with neuropathy. Additionally, one‐way ANOVA and the post‐hoc Bonferroni test were used to assess differences in parametric continuous variables across study groups, while the Kruskal–Wallis test was employed for non‐parametric continuous variables. Pearson correlation was used to analyze the association between continuous variables. The area under the curves for sNGAL, BUN, Creatinine and NLR used in the evaluation of diabetic nephropathy was calculated using the Receiver Operator Characteristics Curve. A *p* value of 0.05 and a 95% confidence interval were used for all statistical significance calculations.

## 3 Results

### 3.1 Baseline Characteristics of Study Participants

The prevalence of nephropathy among type 2 diabetics was 20.6%. The majority of the case group participants enrolled in the study were females, had no formal education, and had an average age of 60 years. Body mass index, fasting blood glucose, systolic blood pressure, glycosylated hemoglobin percent, and creatinine levels showed statistically significant differences (*p* < 0.05) among diabetics with nephropathy (DN), those without nephropathy, and healthy controls. These variables were considerably greater among diabetics with and without nephropathy compared to healthy controls in a post‐hoc Bonferroni test (*p* < 0.05), with the exception of diastolic blood pressure. White blood cell count, absolute neutrophil count, and sNGAL levels were higher in diabetes patients with nephropathy than those without nephropathy and controls (*p* < 0.05). A significant association was found between consumption of sweets, vigorous exercise, and development of nephropathy among study participants (*p* < 0.05) (Table 1).

**Table 1:** Comparison of sociodemographic, anthropometric, hematobiochemical, and dietary lifestyle factors among type 2 diabetics with nephropathy, without nephropathy, and controls.

Variables	Diabetics with nephropathy (*n* = 20)	Diabetics without nephropathy (*n* = 77)	Controls (*n* = 70)	*p* value
Age (yrs)	60.0 (55.3–64.0)	57.0 (51.0–63.0)	45.0 (40.0–50.3)	**< 0.0001** ^**a,b**^
Gender				**0.0340**
Male	5 (25.0)	16 (20.8)	28 (40.0)	
Female	15 (75.0)	61 (79.2)	42 (60.0)	
Marital status				0.2290
Single	10 (50.0)	36 (46.8)	24 (34.3)	
Married	10 (50.0)	41 (53.2)	46 (65.7)	
Residence				**0.0020**
Rural	17 (85.0)	43 (55.8)	29 (41.4)	
Urban	3 (15.0)	34 (44.2)	41 (58.6)	
Educational level				**< 0.0001**
No formal education	15 (75.0)	43 (55.8)	10 (14.3)	
Secondary	2 (10.0)	23 (29.9)	13 (18.6)	
Tertiary	3 (15.0)	11 (14.3)	47 (67.1)	
Weekly vigorous exercise				**< 0.0001**
Rarely	2 (10.0)	4 (5.2)	0 (0.0)	
Irregularly	16 (80.0)	50 (64.9)	10 (14.3)	
Regular	2 (10.0)	23 (29.9)	60 (85.7)	
Monthly sweets consumption				**< 0.0001**
Rarely	10 (50.0)	15 (19.5)	0 (0.0)	
Irregularly	10 (50.0)	62 (80.5)	57 (81.4)	
Regular	0 (0.0)	0 (0.0)	13 (18.6)	
Creatinine (µmol/L)	96.5 ± 31.6	68.1 ± 17.7	52.7 ± 16.0	
BMI (kg/m^2^)	30.3 (26.4–32.3)	28.3 (26.1–30.5)	23.0 (21.8–24.1)	**< 0.0001** ^**a,b**^
FBS (mmol/L)	6.7 (6.5–7.4)	7.6 (6.3–11.3)	4.3 (3.9–4.9)	**< 0.0001** ^**a,b**^
Diastolic (mm/Hg)	67.5 (64.3–76.3)	74.0 (67.5–76.5)	73.0 (72.0–74.0)	0.0700
Systolic (mm/Hg)	126.0 (124.0–128.0)	125.0 (122.0–128.0)	121.0 (119.8–121.0)	**< 0.0001** ^**a,b**^
HbA1c (%)	7.3 (6.8–8.5)	7.1 (6.0–9.0)	5.6 (5.2–6.1)	**< 0.0001** ^**a,b**^
sNGAL (µg/L)	7.5 (5.1–12.3)	5.3 (4.8–5.9)	4.5 (3.6–5.1)	**< 0.0001** ^**a,b**^
BUN (mmol/L)	5.5 (2.6–9.2)	2.2 (1.8–2.5)	2.0 (1.5–2.3)	**< 0.0001** ^**b,c**^
eGFR (mL/min/1.732)	61.5 (51.5–89.8)	93.0 (77.5–104.5)	116.5 (107.0–124.5)	**< 0.0001** ^**a,b,c**^
White blood count (/nL)	10.8 (6.8–15.8)	5.8 (5.1–7.5)	5.8 (5.0–6.5)	**< 0.0001** ^**b,c**^
Absolute neutrophil count (/nL)	8.6 (4.4–13.6)	4.6 (3.9–5.4)	3.9 (3.4–4.6)	**< 0.0001** ^**a,b,c**^
NLR	2.0 (1.6–2.4)	1.9 (1.5–2.6)	1.7 (1.4–2.5)	0.0840

*Note: p *< 0.05 and bolded means statistically significant.

Abbreviations: BMI, body mass index; BUN, blood urea nitrogen; eGFR, estimated glomerular filtration rate; FBS, fasting blood glucose, HbA1c, glycosylated haemoglobin; sNGAL, serum neutrophil gelatinase‐associated lipocalin; UACR, urine albumin‐to‐creatinine ratio.

^a^Significant difference between controls and diabetic nephropathy.

^b^Significant difference between controls and diabetics without nephropathy.

^c^Significant difference between diabetic nephropathy and diabetic without nephropathy UACR.

### 3.2 Univariate and Multivariate Logistic Regression for Risk Factors for Nephropathy in T2DM Patients

In a univariate logistic regression model, compared to participants aged 35–39 years, those aged 40–49 years (crude odds ratio [cOR]: 0.17, 95% CI [0.04–0.82], *p* = 0.027) had significantly lower odds of having diabetic nephropathy. Additionally, individuals residing in urban areas (cOR: 0.17, 95% CI [0.05–0.60], *p* = 0.006), those with secondary (cOR: 0.06, 95% CI [0.01–0.41], *p* = 0.004) and tertiary (cOR: 0.05, 95% CI [0.01–0.32], *p* = 0.001) levels of education, as well as those who consumed sweets irregularly (cOR: 0.13, 95% CI [0.05–0.35], *p* < 0.001) had lower odds of developing nephropathy. Regular vigorous exercise was associated with lower crude odds (cOR: 0.05, 95% CI [0.01–0.44], *p* = 0.007) of diabetic nephropathy; this association was not statistically significant in the adjusted model (aOR = 0.21, *p* = 0.328). Also, gender, marital status, occupation, and fruit and vegetable consumption all had *p* > 0.05 and were excluded from the multivariate logistic regression model analyses.

After adjusting for potential confounders, irregular sweets consumption (adjusted odds ratio [aOR]: 0.11, 95% CI [0.02–0.73], *p* = 0.022) and informal occupation (aOR: 0.01, 95% CI [0.00–0.51], *p* = 0.020) were independently associated with reduced odds of diabetic nephropathy (Table 2).

**Table 2:** Univariate and multivariate logistic regression for risk factors for nephropathy in T2DM patients.

Variable	Diabetics with nephropathy (*n* = 20)	cOR (95% CI)	*p* value	aOR (95% CI)	*p* value
Age category (years)					
35–39	1 (5.0)	1.00	—	1.00	—
40–49	2 (10.0)	0.17 (0.04–0.82)	**0.027**	0.83 (0.00–9374.40)	0.969
50–59	7 (35.0)	0.62 (0.21–1.79)	0.374	1.20 (0.00–13341.70)	0.969
≥ 60	10 (50.0)	0.18 (0.02–1.51)	0.115	0.26 (0.00–2745.72)	0.774
Gender					
Male	5 (25.0)	1.00	—	1.00	—
Female	15 (75.0)	1.28 (0.44–3.74)	0.650	0.51 (0.08–3.34)	0.484
Marital status					
Single	10 (50.0)	1.00	—	1.00	—
Married	10 (50.0)	0.69 (0.27–1.76)	0.437	1.23 (0.20–7.66)	0.822
Residence					
Rural	17 (85.0)	1.00	—	1.00	—
Urban	3 (15.0)	0.17 (0.05–0.60)	**0.006**	0.31 (0.05–2.01)	0.216
Educational level					
No formal education	4 (20.0)	1.00	—	1.00	—
Basic	11 (55.0)	0.22 (0.05–1.04)	0.056	0.30 (0.02–4.51)	0.385
Secondary	2 (10.0)	0.06 (0.01–0.41)	**0.004**	0.18 (0.01–7.06)	0.358
Tertiary	3 (15.0)	0.05 (0.01–0.32)	**0.001**	0.06 (0.00–2.30)	0.128
Occupation					
Retired	2 (10.0)	1.00	—	1.00	—
Unemployed	9 (45.0)	1.69 (0.30–9.36)	0.549	0.17 (0.01–2.15)	0.169
Informal	5 (25.0)	0.28 (0.05–1.65)	0.158	0.01 (0.00–0.51)	**0.020**
Formal	4 (20.0)	0.55 (0.09–3.47)	0.521	0.22 (0.01–6.30)	0.379
Weekly vigorous exercise					
Rarely	2 (10.0)	1.00	—	1.00	—
Irregularly	16 (80.0)	0.53 (0.09–3.18)	0.49	3.34 (0.22–51.09)	0.387
Regular	2 (10.0)	0.05 (0.01–0.44)	**0.007**	0.21 (0.01–4.75)	0.328
Monthly sweets consumption					
Rarely	10 (50.0)	1.00	—	1.00	—
Irregularly	10 (50.0)	0.13 (0.05–0.35)	**< 0.001**	0.11 (0.02–0.73)	**0.022**
Regular	0 (0.0)	*	0.999	*	0.998
Fruits and vegetable consumption					
Rarely	1 (5.0)	1.00	—	1.00	—
Irregularly	7 (35.0)	0.20 (0.02–2.53)	0.215	0.98 (0.04–26.52)	0.990
Regular	12 (60.0)	0.32 (0.03–3.76)	0.362	8.90 (0.29–277.30)	0.213

*Note: p* < 0.05 and bolded means statistically significant. For categories marked with *, no odds ratio or CI is provided due to insufficient data.

Abbreviations: aOR, adjusted odds ratio; CI, Confidence interval; cOR, crude odds ratio.

### 3.3 The Distribution and Comparison of sNGAL and NLR Between Healthy Controls, and Good and Poor Glycemic Controlled Diabetics

**Figure 1:**
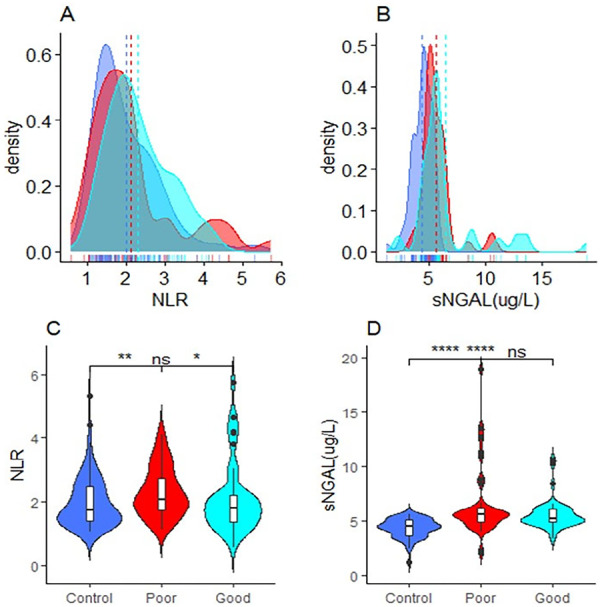
Density (A, B) and box and violin plot (C, D) showing the distribution and comparison respectively of NLR, and sNGAL between good and poor glycaemic controlled type 2 diabetics with nephropathy and healthy controls; ns (not significant) = *p* > 0.05, * = *p* < 0.05, ** = *p* < 0.01, **** = *p* < 0.0001.

Out of the 20 diabetic nephropathy patients, 8 (40%) were good glycemic controlled and 12 (60%) were poor glycemic controlled. We observed a significantly higher NLR in poor glycemic controlled DN than good glycemic control and healthy subjects [2.09 (1.73–2.78) vs. 1.81 (1.36–2.22) vs. 1.72 (1.40–2.51) respectively, (*p* < 0.05)]. sNGAL level was also significantly higher among DN patients with poor and good glycemic control than the healthy individuals [5.60 (4.8–6.20) vs. 5.18 (4.88–6.11) vs. 4.50 (3.60–5.10)] µg/L respectively, (*p* < 0.05)]. NLR levels between good glycaemia control and controls, as well as sNGAL levels between poor and good glycemic controlled DN were not statistically significant (*p* > 0.05) (Figure 1).

### 3.4 Pearson Correlations Between BUN, eGFR, and **UACR** in Good and Poor Glycaemic Controlled Diabetics

**Figure 2:**
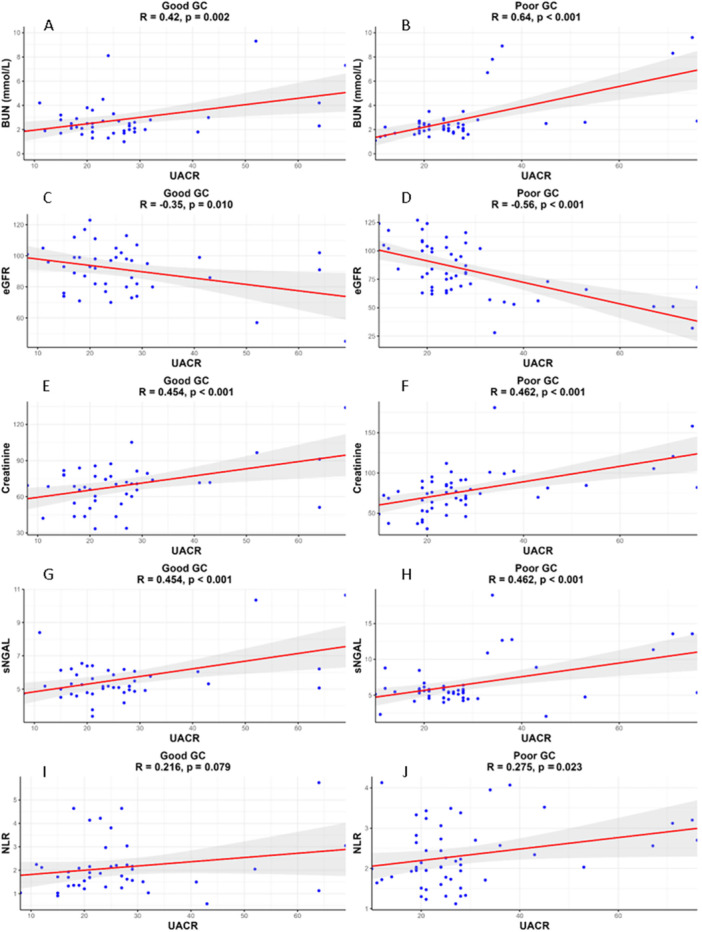
Relationship between UACR and various renal markers (BUN, eGFR, Creatinine, sNGAL and NLR) in type 2 diabetics with good and poor glycaemic control (A–J).

Among DN patients with good glycemic control, there was a moderate positive correlation between UACR and BUN *r* = 0.42, *p* = 0.002 (Figure 2A), and a moderate negative correlation between UACR and eGFR *r* = −0.35, *p* = 0.010 (Figure 2C). UACR was also significantly and positively correlated with creatinine (*r* = 0.454, *p* < 0.001 (Figure 2E) and sNGAL *r* = 0.454, *p* < 0.001 (Figure 2G). The correlation between UACR and NLR was weaker and not statistically significant *r* = 0.216, *p* = 0.079 (Figure 2I).

In contrast, among DN patients with poor glycemic control, UACR was strongly and positively correlated with BUN *r* = 0.64, *p* < 0.001 (Figure 2B), and moderately negatively correlated with eGFR *r* = −0.56, *p* < 0.001 (Figure 2D). Positive correlations were also found between UACR and creatinine *r* = 0.462, *p* < 0.001 (Figure 2F), sNGAL *r* = 0.462, *p* < 0.001 (Figure 2H), and NLR *r* = 0.27, *p* = 0.023 (Figure 2J), all of which were statistically significant.

### 3.5 Diagnostic Performance of NGAL and NLR

**Figure 3:**
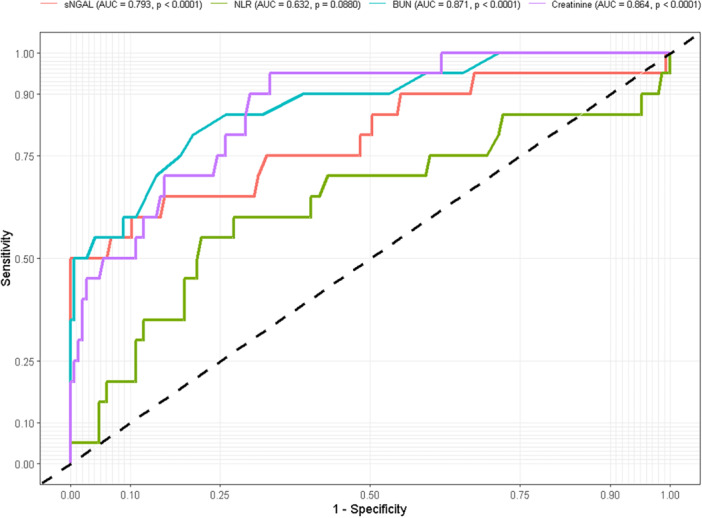
The receiver operating characteristics (ROC) curves of sNGAL, BUN, creatinine and NLR for predicting diabetic nephropathy among T2DM.

Receiver operating characteristic (ROC) analysis showed that sNGAL had a good performance (AUC = 0.793, *p* < 0.001) and NLR had a moderate performance (AUC = 0.632, *p* = 0.082) for predicting diabetic nephropathy (Figure 3). An sNGAL cut‐off of 8.87 µg/L had 80.0% sensitivity (95% CI: 28.1–71.9) and 100.0% specificity (95% CI: 100.0–100.0), with a positive predictive value (PPV) of 100.0% and a negative predictive value (NPV) of 93.6%. An NLR threshold of 2.34 had 60.0% sensitivity (95% CI: 38.5–81.5) and 72.8% specificity (95% CI: 65.6–80.0), with a PPV of 23.1% and an NPV of 93.0% in predicting nephropathy in type 2 diabetes mellitus patients. Moreover, BUN (AUC = 0.871, *p*< 0.0001) and creatinine (AUC = 0.864, *p*< 0.0001), demonstrated good diagnostic performance for predicting diabetic nephropathy. At a cut‐off of 69.4 µmol/L, creatinine showed 55.0% sensitivity (95% CI: 34.2–74.1) and 79.6% specificity (95% CI: 72.3–85.3). Also, at a cut‐off of 2.50 mmol/L, BUN had 50.0% sensitivity (95% CI: 30.0–70.0) and 66.7% specificity (95% CI: 58.7–73.8) (Table 3).

**Table 3:** Diagnostic performance of sNGAL and NLR in predicting diabetic nephropathy among type 2 diabetics.

Marker	Cut‐off	Sensitivity (95% CI)	Specificity (95% CI)	PPV	NPV	LR +	LR−
sNGAL (µg/L)	8.87	80.0 (57.7–92.3)	100.0 (100.0–100.0)	100.0	93.6	Inf	0.5
NLR	2.34	60.0 (38.5–81.5)	72.8 (65.6–80.0)	23.1	93	2.2	0.55
BUN	2.50	50.0 (30.0–70.0)	66.7 (58.7–73.8)	34.8	96.7	3.92	0.25
Creatinine	69.4	55.0 (34.2–74.1)	79.6 (72.3–85.3)	27.9	99	2.85	0.08

Abbreviations: CI, Confidence interval; LR+, positive likelihood ratio; LR−, negative likelihood ratio; NLR, neutrophil‐to‐lymphocyte ratio; NPV, negative predictive value; PPV, positive predictive value; sNGAL, serum neutrophil gelatinase‐associated lipocalin.

### 3.6 Predictors of Nephropathy in Type 2 Diabetes Mellitus

In the univariate binary logistic regression model, higher levels of sNGAL (cOR 2.04, 95% CI: 1.49–2.77, *p* < 0.001) were strongly associated with more than two‐fold increase in risk of nephropathy in patients with type 2 diabetes mellitus. Similarly, higher NLR was moderately linked to nephropathy (cOR 1.64, 95% CI: 1.05–2.57, *p* = 0.031). Creatinine demonstrated a modest association, with a slight but statistically significant increase in risk as levels rose (cOR 1.08, 95% CI: 1.04–1.11, *p* < 0.001). BUN (cOR 2.22, 95% CI: 1.58–3.13, *p* < 0.001) increased the chance of having nephropathy in patients with type 2 diabetes mellitus by more than two folds.

In the multivariate binary logistic regression model, after adjusting for all included biomarkers, sNGAL remained a significant predictor of nephropathy in patients with type 2 diabetes mellitus. sNGAL showed a modest independent association, with higher levels linked to a slight but statistically significant increase in risk (aOR 1.25, 95% CI: 0.71–2.21, *p* = 0.014). In contrast, NLR was not significantly associated with nephropathy in the adjusted model (NLR: aOR 1.16, 95% CI: 0.60–2.24, *p* = 0.663). Creatinine showed a modest association, with higher levels linked to a slight but statistically significant increase in risk (aOR 1.05, 95% CI: 1.01–1.09, *p* = 0.038). Also, BUN had an insignificant association, with increased levels associated with higher odds of nephropathy (aOR 1.63, 95% CI: 1.03–2.59, *p* = 0.444) (Table 4).

**Table 4:** sNGAL and NLR as predictors of nephropathy in type 2 diabetes mellitus.

Predictor	cOR (95% CI)	*p* value	aOR (95% CI)	*p* value
sNGAL (µmol/L)	2.04 (1.49–2.77)	**< 0.001**	1.25 (0.71–2.21)	**0.014**
NLR	1.64 (1.05–2.57)	**0.031**	1.16 (0.60–2.24)	0.663
Creatinine (µmol/L)	1.08 (1.04–1.11)	**< 0.001**	1.05 (1.01–1.09)	**0.038**
BUN (mmol/L)	2.22 (1.58–3.13)	**< 0.001**	1.63 (1.03–2.59)	0.444

*Note:* Bold *p* values indicate statistical significance at *p* < 0.05.

Abbreviations: aOR, adjusted odds ratio from multivariate binary logistic regression; CI, confidence interval; cOR, crude odds ratio from univariate binary logistic regression; NLR, neutrophil‐to‐lymphocyte ratio; sNGAL, serum neutrophil gelatinase‐associated lipocalin.

## 4 Discussion

Effective monitoring is paramount to preventing the exacerbation of diabetes mellitus to DN. This can be achieved by identifying biomarkers that can provide an early indication of renal impairment as well as markers that are readily available and less expensive. This hospital‐based case‐control study aimed to investigate the diagnostic value of sNGAL and NLR for the detection of nephropathy among type 2 diabetics.

In the present study, age was comparable among patients with DN and without DN, but there was a statistical difference in age and gender when all diabetic patients were compared to non‐diabetic patients. Multivariate logistic regression model analyses revealed that age and gender are not determinant of nephropathy in diabetic patients ruling out bias in participants selection. This is consistent with a study conducted by Mengistu et al. [24] but inconsistent with findings from Hintsa et al. [25]. In our study, exercise did not exert an independent effect on the development of nephropathy. This is consistent with a study conducted by He et al. [26], who found no correlation between exercise and diabetic nephropathy.

The prevalence of DN among T2DM patients from this present study was significant (20.6%) and reflective of findings by Tannor et al. and Ofori et al. [6, 27], which reported between them 16.1% to 19% prevalence of CKD among diabetes mellitus patients in Ghana. The prevalence observed in this study is alarming and could be attributed to patients not taking their medications as directed by physicians or the therapy regimen not working for them.

In this study, the NLR level was insignificantly higher in DN patients than in those without DN, inconsistent with findings from Paulus et al. [28], who reported a significant increase in NLR levels among DN patients than those without DN. This could be attributed to excess glucose in circulation, causing damage to the renal tubules, subsequently inducing a persistent inflammatory state. Moreover, studies conducted by Wan et al. [29] reported comparable levels of NLR in DN patients and without DN, which is in line with our findings. The reason for this inconsistency includes differences in sample size, population and geography. In addition, a significant increase in NLR was found in DN patients with poor glycemic control than those with good glycemic control and this is reflective of the findings from Hussain et al. [30] that suggested that an elevated HbA1c is associated with high NLR levels due to an induced chronic inflammatory state by the hyperglycemic milieu.

sNGAL levels in this study were found to be significantly higher in DN patients than in those without DN. This could be due to damage to renal tubules as a result of the presence of excess glucose in circulation, leading to endothelial dysfunction and tubulointerstitial damage consistent with findings from Kaul et al. [31]. Furthermore, a mild rise in levels of sNGAL level was also found in DN with both poor and good glycemic control than apparently healthy non‐diabetics which also agrees with Kaul et al. [31] that sNGAL is an early predictor of DN in type 2 diabetics. This explains why even in good glycemic controlled diabetics, levels of sNGAL were mildly increased.

Moreover, consistent with findings from Wu et al. [32], sNGAL had a moderately positive correlation with UACR in both good and poor glycemic control diabetic patients, with a slightly higher strength of relationship in poor glycemic control diabetic patients. In the same vein, NLR also had a weak significant positive correlation with UACR, which agrees with a study conducted by Mohamed et al. [33]. This may be attributed to the fact that diabetic patients are in their initial stages of developing nephropathy.

This study also examined the diagnostic performance of these markers as indicators of DN. The excellent cut point to indicate DN was assessed using microalbuminuria as a reference. This study found NGAL µg/L (AUC = 0.793) to be a good indicator of DN. These findings are consistent with previous studies which observed good performance of these markers for diagnosing DN [18, 22, 23, 24]. sNGAL was highly sensitive and specific, which is in line with findings of Kaul et al. [31] (sensitivity 95.1%; specificity 100%) and Danquah et al. [34] (sensitivity 100.0%; specificity 72.0%). The sensitivity and specificity of sNGAL in this study vary from what has been reported in some previous studies, likely reflecting differences in study population characteristics, inclusion criteria, and cut‐off selection. The sensitivity of NGAL reported in this study was low compared to the aforementioned studies and this could be due to participants being at the early stage of CKD. Moreover, NLR with an insignificant AUC of 0.632 performed poorly in this study. The sensitivity and specificity values for NLR were different from reports of Singh et al. [24] (sensitivity 89.7%, specificity 69.7%) and findings of Wan et al. [29] (sensitivity 68.0%, specificity 47.8%). These slight variations could be due to ethnic or geographical differences between the populations of study. These findings from the present study, however, suggest that chronic inflammation is involved in the development of kidney disease in type 2 diabetics [35, 36].

Interestingly, sNGAL was found to be an independent predictor of diabetic nephropathy in both univariate and multivariate binary logistic regression models, making NGAL an ideal marker for assessing kidney diseases at early stages. This is in line with the findings from Danquah et al. [34], which reported that plasma NGAL was a reliable indicator of kidney damage in the initial phases of CKD. This indeed makes sNGAL a good marker for kidney injury as even before glycemic control worsens. However, NLR couldn't predict nephropathy in the multivariate analysis, and this is consistent with a similar study conducted by Upadhyayula et al. [37], who also found that NLR is not an independent predictor of nephropathy in multivariate analysis. This may be due to the fact that our patients were at the initial stages of kidney diseases which may influence their overall inflammatory status.

With regards to the traditional markers, creatinine and BUN showed moderate performance when compared to sNGAL [38]. In the regression analysis, Creatinine showed a significant association in both the univariate and multivariate models. BUN, on the other hand, showed significant increased odds in the univariate model but had an insignificant increased odds of predicting DN [39]. Commenting on the diagnostic performance, Creatinine and BUN showed a significant ROC performance with higher AUC values even more than sNGAL, but their sensitivities and specificities were low as compared to sNGAL. Overall, while sNGAL demonstrated clear superiority as an independent early predictor of diabetic nephropathy, our findings indicate that traditional markers such as creatinine and BUN also showed good diagnostic performance and remain valuable tools that should not be overlooked in clinical practice [40, 41].

The strength of this study is that, it is the first attempt to investigate the diagnostic value of sNGAL and NLR for the detection of nephropathy in Ghanaian type 2 diabetes mellitus patients. This may help inform local clinical decision‐making and guide future multicenter studies aimed at validating sNGAL and NLR in broader and more diverse cohorts. Despite these findings, this study has certain limitation. This study was a one‐center study and may not be a true reflection of the entire Ghanaian population. Another limitation is the small sample size, but the results from this study provide a basis for understanding of nephropathy markers in Ghana and could be used as a reference study for further research in Ghana. Although urine cultures were performed to exclude urinary tract infections and strict clinical and hematological criteria were applied, no serum inflammatory markers (e.g., CRP, procalcitonin) were measured. As such, subclinical infections at other sites cannot be fully ruled out. Hence, further studies should classify the overall inflammatory status of diabetic patients using specific serum inflammatory markers. We also recommend that larger prospective studies integrating NLR with established clinical and laboratory markers may clarify whether it has any incremental value as part of a composite risk model. Although hypertensive individuals and those with advanced kidney disease were excluded, we did not collect data on the use of medications that may influence proteinuria, which could represent a residual confounding factor.

## 5 Conclusion

In conclusion, sNGAL, as an independent biomarker, is an excellent indicator of kidney disease as levels increases in both good and poor glycemic controlled type 2 diabetics with nephropathy. NLR should not be used as a standalone indicator of diabetic nephropathy.

## Author Contributions

**Allwell Adofo Ayirebi:** conceptualization, investigation, writing – original draft, methodology, writing – review and editing, project administration, resources, supervision, software, data curation, visualization. **Wina Ivy Ofori Boadu:** conceptualization, data curation, supervision, validation, writing – review and editing. **Stephen Twumasi:** conceptualization, data curation, formal analysis, investigation, methodology, software, writing – original draft, writing – review and editing. **Samuel Kwarteng:** formal analysis, writing – original draft, writing – review and editing. **Benedict Sackey:** data curation, project administration, visualization, writing – review and editing. **Lilian Antwi Boateng:** data curation, writing – review and editing. **Joseph Boachie:** data curation, writing – review and editing. **Samuel Essien‐Baidoo:** writing – review and editing. **Daniel Nii Martey Antonio:** data curation, investigation, methodology, writing – review and editing. **John Agyemang Sah:** data curation, writing – review and editing. **Afua Marfowaa:** writing – review and editing, data curation. **Mayfair Adwapa Mpiani:** data curation, writing – review and editing. **Joachim Baba Domosie:** data curation, writing – review and editing. **Abiba Khalifah:** writing – review and editing. **Emmanuel Ekow Korsah:** formal analysis, writing – review and editing. **Ebenezer Senu:** formal analysis, writing – review and editing. **Eugene Arele Ansah:** formal analysis, writing – review and editing, Methodology. **Joseph Yorke:** writing – review and editing. **Enoch Odame Anto:** conceptualization, project administration, supervision, validation, visualization, writing – original draft, writing – review and editing.

## Funding

The authors received no specific funding for this word.

## Conflicts of Interest

The authors declare no conflicts of interest.

## Transparency Statement

The corresponding authors, Allwell Adofo Ayirebi, Wina Ivy Ofori Boadu, Stephen Twumasi, and Enoch Odame Anto, affirm that this manuscript is an honest, accurate, and transparent account of the study being reported; that no important aspects of the study have been omitted; and that any discrepancies from the study as planned (and, if relevant, registered) have been explained.

## Data Availability

The authors confirm that the data supporting the findings of this study are available within the article. Data and materials for study are available upon request from the corresponding authors.
